# Rapid Diagnosis of *Mycobacterium tuberculosis* with Truenat MTB: A Near-Care Approach

**DOI:** 10.1371/journal.pone.0051121

**Published:** 2013-01-21

**Authors:** Chaitali Nikam, Manjula Jagannath, Manoj Mulakkapurath Narayanan, Vinaya Ramanabhiraman, Mubin Kazi, Anjali Shetty, Camilla Rodrigues

**Affiliations:** 1 Department of Microbiology, P. D. Hinduja Hospital and Medical Research Centre, Mahim, Mumbai, India; 2 bigtecLabs, bigtec Pvt.Ltd, Rajajinagar, Bangalore, India; Institut de Pharmacologie et de Biologie Structurale, France

## Abstract

**Background:**

Control of the global Tuberculosis (TB) burden is hindered by the lack of a simple and effective diagnostic test that can be utilized in resource-limited settings.

**Methods:**

We evaluated the performance of Truenat MTB™, a chip-based nucleic acid amplification test in the detection of *Mycobacterium tuberculosis* (MTB) in clinical sputum specimens from 226 patients with suspected pulmonary tuberculosis (TB). The test involved sputum processing using Trueprep-MAG™ (nanoparticle-based protocol run on a battery-operated device) and real-time PCR performed on the Truelab Uno™ analyzer (handheld, battery-operated thermal cycler). Specimens were also examined for presence of MTB using smear microscopy, liquid culture and an in-house nested PCR protocol. Results were assessed in comparison to a composite reference standard (CRS) consisting of smear and culture results, clinical treatment and follow-up, and radiology findings.

**Results:**

Based on the CRS, 191 patients had “Clinical-TB” (Definite and Probable-TB). Of which 154 patients are already on treatment, and 37 were treatment naïve cases. Remaining 35 were confirmed “Non-TB” cases which are treatment naïve cases. The Truenat MTB test was found to have sensitivity and specificity of 91.1% (CI: 86.1–94.7) and 100% (CI: 90.0–100) respectively, in comparison to 90.58% (CI: 85.5–94.3) and 91.43% (CI: 76.9–98.2) respectively for the in-house nested PCR protocol.

**Conclusion:**

This preliminary study shows that the Truenat MTB test allows detection of TB in approximately one hour and can be utilized in near-care settings to provide quick and accurate diagnosis.

## Introduction

Tuberculosis (TB) causes the highest number of deaths globally, attributable to a curable infectious agent [1], despite the availability of potent anti-TB medication. Reduction in TB-related morbidity and mortality is impeded by the lack of rapid and cost-effective diagnostic tests that are implementable in resource-limited settings. Over 95% of new TB cases and TB deaths occur in developing countries [2], where smear microscopy, which detects only 45% of TB infections [3], remains the most practical and often only test available.

Even where infrastructure exists, more sensitive tests are currently time- and cost-prohibitive. Performing a culture can take weeks because of the slow growth rate of TB bacilli. Molecular tests such as polymerase chain reaction (PCR), which are considerably faster than culture, often have a high turnaround time as specimens are often sent to distant laboratories. The expense involved in PCR testing makes it out of reach of most patients in TB-endemic countries. High risk of transmission of TB makes cost-effective and rapid detection crucial to control the spread of infection.

There has been considerable interest in the miniaturization of the PCR platform as this would confer advantages such as reduction in cost of instruments and tests, faster turnaround times and enhancement in the availability and accessibility of PCR tests in resource-poor geographies. With the combined advantages of affordability, simplicity in operations, diagnostic sensitivity and portability, micro-PCR devices are strong candidates for wide-scale use among the peripheral laboratories of India and other countries of South-East Asia which account for 50% of the global burden of MTB [4].

In this study, we evaluate a novel TB test, Truenat MTB (bigtec Labs, India). The test requires the user to add 5 µl of extracted DNA to a pre-loaded microchip [5] containing room temperature stabilized reagents and start the PCR run on a handheld battery-operated device, Truelab Uno™, which is a fully portable stand-alone thermal cycler [6]. Briefly, the Truelab platform consists of a PDA (personal digital assistant) running the software application, a handheld unit housing the control electronics and optical detection system for real-time monitoring and a microchip with integrated temperature control elements. The Truenat MTB test involves sputum processing using a battery-operated sample preparation device, Trueprep-MAG™, which extracts nucleic acids by a simple menu driven process using a nanoparticle-based protocol optimized for sputum. The device integrates all operations (heating, fluid mixing, magnet control, step timing) using on a programmed micro-controller, and easy to follow screen instructions, thereby enabling nucleic acid isolation without the need for any additional equipment. The chip-based test has been designed to simplify the process of real-time PCR from ‘sample to result’ so that laboratories with minimal infrastructure can easily perform these tests routinely in their facilities and report PCR results in less than an hour.

## Materials and Methods

### Ethics

This study was approved by the Institutional Review Board of Hinduja hospital. Waiver of consent was obtained by Institutional Review Board, PD Hinduja Hospital and MRC., Mumbai, India. Waiver of consent was obtained as the study was carried out on left-over banked sediments identified by a laboratory generated number with no traceability to the patients. All patients' details were thus kept confidential. The Truenat MTB results were not used in clinical decision making.

### Settings

Sample collection, Smear Microscopy, MGIT culture and nested PCR was performed at Hinduja Hospital and Medical Research Centre, Mumbai. The Truenat MTB tests were performed by Hinduja staff at bigtec Laboratories, Bangalore.

### Study population and specimens

**Figure 1:**
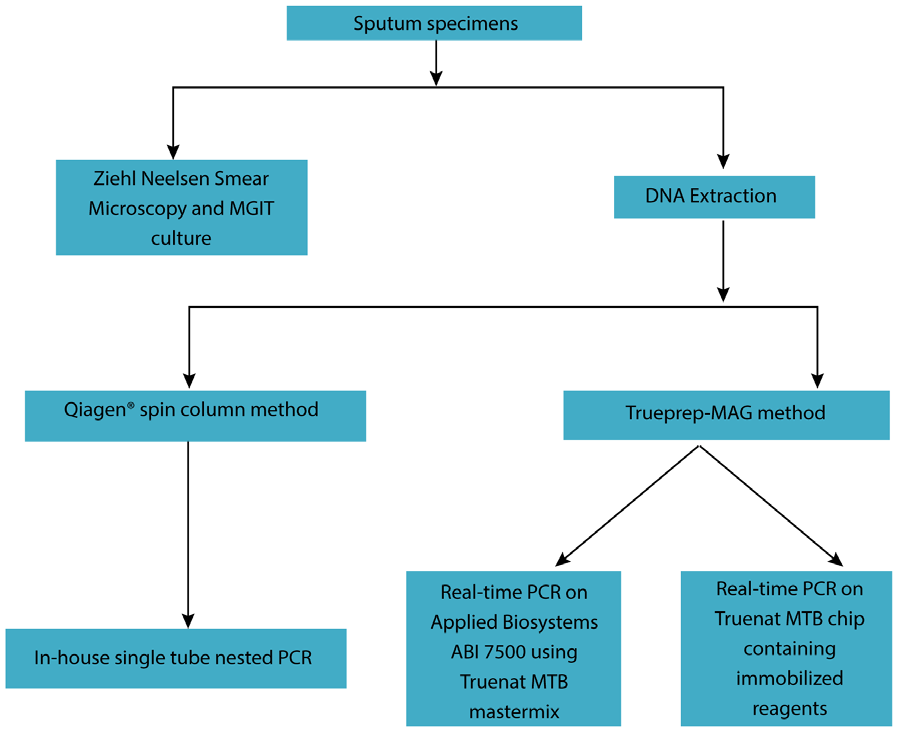
Study design for evaluation of Truenat MTB performance.

This was a single site, blinded, cross-sectional study to determine the performance of the Truenat MTB in patients with symptoms of pulmonary TB in comparison to conventional methodologies. Sputum specimens were taken from patients presenting routinely to our hospital with suspected pulmonary TB. Standard diagnostic follow-up (smear, culture, and in-house nested PCR) was performed on all patients. Where available, left-over sputum specimens were tested using Truenat MTB. This study was approved by the Institutional Review Board of Hinduja hospital. (Fig. 1)

### Methods

As described previously [7], direct and concentrated acid-fast bacillus (AFB) microscopy (Ziehl-Neelsen [ZN] staining) was performed, followed by sputum processing with 2% N-acetyl-L-cysteine and sodium hydroxide (NALC-NaOH) and centrifugation. The re-suspended pellet was subjected to cultivation on liquid medium (MGIT [mycobacteria growth indicator tube]).

Digested and decontaminated (2% NALC-NaOH) sputum specimens that were culture negative for mycobacterium and confirmed “Non-TB cases” were pooled for use as a negative control. A suspension of *M. tuberculosis* H37RV was prepared in sterile saline and adjusted to the density of a 1.0 McFarland standard. The suspension was diluted 1∶10 in saline and used to spike the pooled above mentioned negative control and used as a positive control. Spiked specimens were stored at −70°C until further processing.

### Patient categories

A composite reference standard (CRS) was used to categorise patients. Patients were allocated into the following groups based on a combination of smear status, culture results, clinical treatment and follow-up, and radiology findings. As per routine hospital protocol, a follow-up was performed on all patients based on their culture results which were available 4–6 weeks after inoculation. Definite-TB: Smear positive and Culture positive (S+C+) or smear negative, culture positive (S−C+)

#### Probable-TB

Smear positive and culture negative(S+C−) Smear negative and Culture negative(S−C−) but clinical-radiological picture highly indicative of TB. Patients showing a response to anti-TB treatment at follow-up were assigned to this group. During the follow up, the following queries were addressed to patients: whether empirical anti-TB Treatment was initiated at a previous visit(s); whether the patient had a previous history of TB;, whether the patient had contact history Of TB; supportive findings of TB in any another clinical tests, e.g. Radiology. Patients who responded “yes” to any of the queries or had supportive findings indicative of TB were included in this group.

#### Non-TB

Smear negative culture negative (S−C−) Patient was assessed as ‘clinically negative’ when the patient had no previous history of TB infection and no microbiological evidence indicating current infection. Patients were symptomatic (weight loss and prolonged cough) but showed improvement without anti-TB treatment.

### Nested PCR

#### 1. Nucleic acid extraction

Untreated sputum specimens were processed using the Qiagen QIAamp DNA mini kit (Qiagen) with the following modifications: 1) 30 mg/ml lysozyme (Amresco) was added to the sputum specimen which was then kept at 37°C overnight to ensure enzymatic digestion 2) This was followed by addition of 250 µl lysis buffer (AL buffer from the QIAamp kit) and 25 µl Proteinase K and incubation at 56°C for 2 hours. DNA was then extracted as per manufacturer's recommendations in a total volume of 100 µl.

#### 2. PCR

The multi-copy IS6110 sequence was selected as the target region for the nested PCR test (Table 1). The outer primers, INSF and INSR, were used to amplify a 245 bp fragment. IS1 and IS2 were used as inner primers for the nested PCR, yielding a 123 bp fragment. Primers were purchased from Sigma. The in-house nested PCR was performed as described previously [8]. Briefly, 20 µl of extracted DNA was added to 80 µl of mastermix to bring the total reaction volume to a 100 µl. PCR was run on the Eppendorf Mastercycler gradient. Amplicons was detected by gel electrophoresis using a 3% agarose gel. An internal amplification control, β-globin, was used to detect PCR inhibition in extracts. In case of inhibition the PCR, was repeated after dilution of DNA in a 1∶1 ratio with sterile water.

**Table 1:** Primers used for nested PCR.

Primer	Region	Product size	Nucleotides
Forward:5′CGTGAGGGCATCGAGGTGGC3′	INS	245 bp	631–650
Reverse:5GCGTAGGCGTCGGTGACAAA3′			856–875
Forward:5′CTCGTCCAGCGCCGCTTCGG3′	IS	123 bp	762–781
Reverse:5′CCTGCGAGCGTAGGCGTCGG3′			865–884
Forward:5′TGAACGTGGATGAAGTTGGTGGTG3′	β-globin	291 bp	-
Reverse:5′ACTTTCTTGCCATGAGCCTTCACCTT3			

### Truenat MTB test

#### 1. DNA extraction using Trueprep-MAG protocol

**Figure 2:**
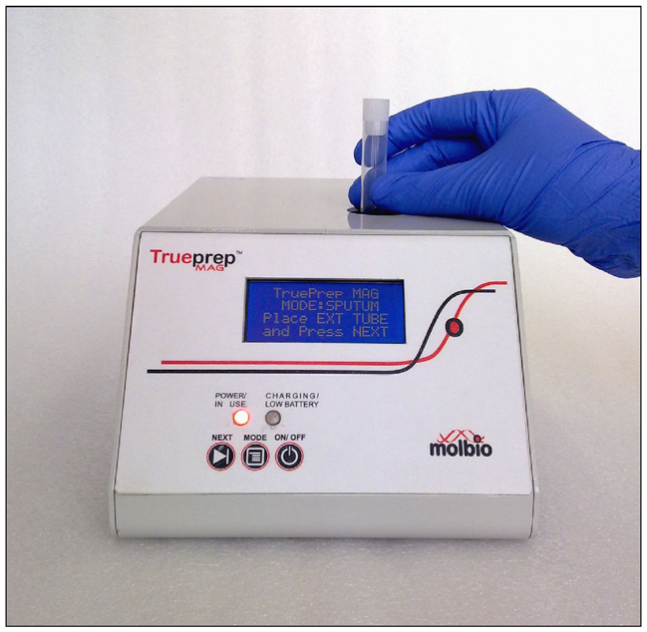
Sample loading on Trueprep-MAG device.

Untreated sputum specimens were liquefied with 500 µl of liquefaction buffer. The liquefied sputum was centrifuged at 14000 rpm for 5 minutes. After discarding supernatant, sputum pellets were re-suspended with 500 µl of lysis buffer and the suspension was incubated at 90°C for 10 minutes in the extraction tube (Fig. 2).

After the lysis step, 500 µl of Binding Reagent A and 100 µl of Binding Reagent B were added to the extraction tube. The bound DNA was washed once with 1 ml of Wash Buffer A and 5 times with 1 ml of Wash Buffer B. 100 µl of elution buffer was added to the tube followed by incubation at 90°C for 5 minutes. Elute was used directly in PCR reactions.

#### 2. Real-time PCR on ABI 7500

PCR reactions were run using the DNA extracted using the Trueprep-MAG protocol. 4 µl of extracted DNA was mixed with 6 µl of the Truenat MTB mastermix and real-time PCR was performed on ABI 7500 (Applied Biosystems) under the following cycling conditions: 1 min at 95°C and 45 cycles of 10 s at 95°C and 34 s at 58°C.

#### 3. Real-time PCR on chip

**Figure 3:**
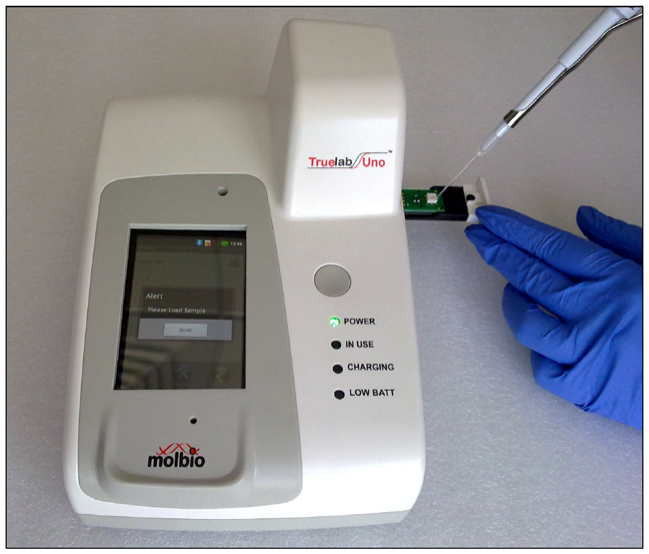
Addition of 5 µl of DNA to Truenat MTB chip.

5 µl of DNA extracted added to the Truenat MTB microchip (Fig. 3) and the real-time PCR was done using a pre-programmed profile on the device. Results were observed on the screen and compared to the results obtained on the ABI 7500 using the same mastermix.

#### 3. Buffers, reagents and mastermixes

All buffers and reagents used for nucleic acid extraction and all mastermixes used for PCR are proprietary components of the Truenat MTB kit.

### Statistical Analysis

Evaluation of the Truenat MTB test was performed done in comparison to the other molecular methods for detection of *Mycobacterium tuberculosis* DNA from sputum, following the STARD recommendations [9]. Sensitivity, Specificity, Positive Predictive Value, Negative Predictive Value, Positive Likelihood Ratio, Negative Likelihood Ratio were calculated by using Bayesian sensitivity/specificity calculator and ROC curve and forest plot were calculated using Meta disc (version 1.4).

## Results

**Figure 4:**
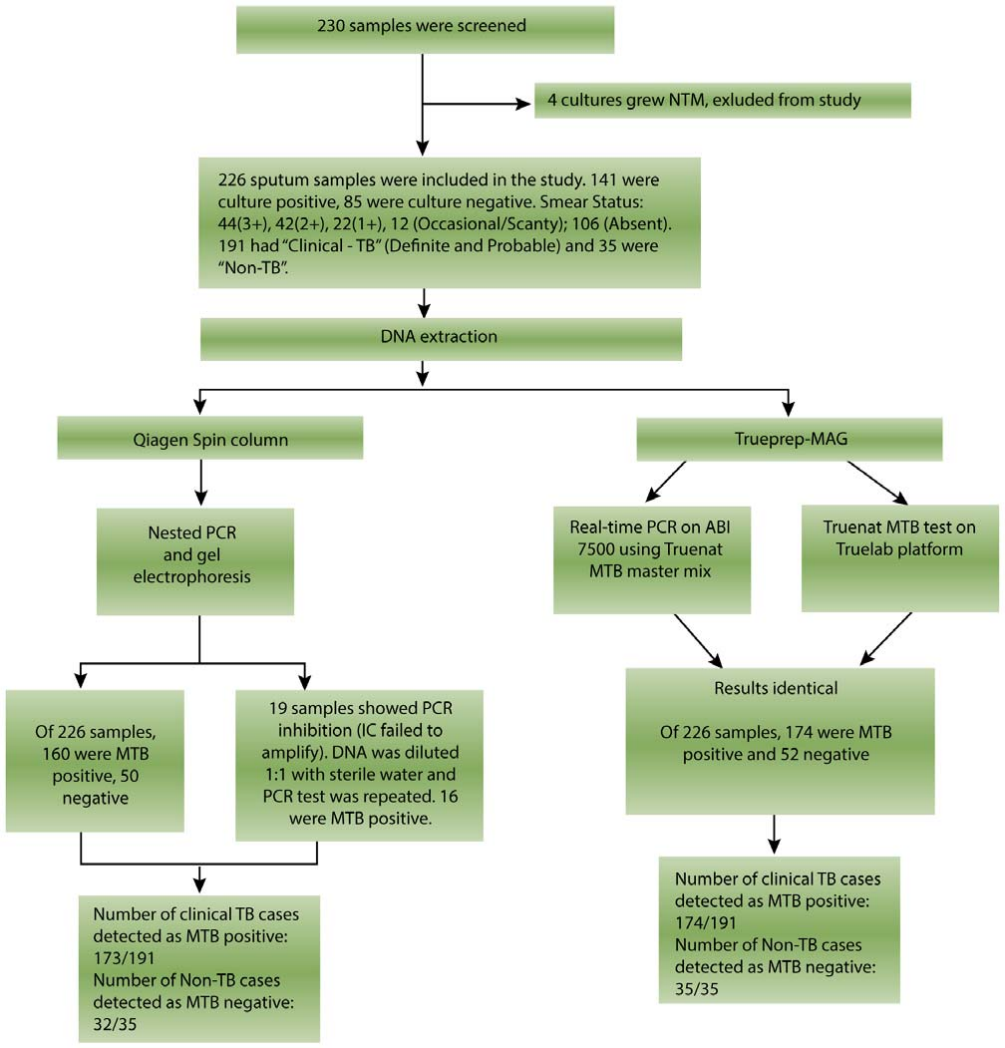
Enlistment and outcome of study.

As shown in fig. 4, outcome of study out of total 230 specimens screened, 4 were detected as nontuberculous mycobacteria (NTM) by phenotypic MGIT and hence were excluded from this study. Of the remaining 226 sputum specimens, 141 were MTB culture positive(C+) and 85 were culture negative(C−). Out of 141 C+, 104 patiets were on antitubercle treatment and 37 were treatment naïve, out of 85 C−, 50 patients were on treatment and 35 were treatment naïve. A total of 112 specimens were smear positive (S+). Among the S+ specimens and 104 (93.33%) were C+ and 8 S+ specimens failed to grow in culture medium. Of the 8 S+C−, 3 had a smear status of 3+, 2 were 2+, 2 were 1+ and 1 had occasional AFB (acid fast bacilli). These S+C− specimens were CRS+ and therefore the inhibition rate for liquid culture among S+ specimens was 6.67% (8/120). As seen in Table 2, of the 120 S+, 117 (97.50%) were detected as positive by the IS6110 nested PCR, and 119 (99.16%) were detected as positive by the Truenat MTB test. One sample with smear status 3+ showed inhibition and was MTB negative by all molecular methods. Of 141 C+ specimens, 135 (95.74%) were detected as positive by the IS6110 nested PCR, and 132 (93.62%) were detected as positive by the Truenat MTB test.

**Table 2:** Performance of PCR tests in various patient groups.

	Smear		Culture		S+C+(n = 112)	S−C−(n = 77)
Truenat MTB	+	−	+	−		
+	119	55	132	42	111	34
−	1	51	9	43	1	43
In-house nested PCR						
+	117	59	135	41	111	35
−	3	47	6	44	1	42

Among the S+C+ specimens, both the in-house nested PCR and Truenat MTB detected 99.12% (111/112) of specimens accurately Among the S−C+ specimens, 75.86% (22/29) were Truenat MTB positive and 82.76% (24/29) were positive by the IS6110 nested PCR protocol.

The Truenat MTB results were largely concordant with the in-house nested PCR results, 196 of 226 specimens showed the same result by either PCR test (Table 3). Of the 30 discordant results, 16 specimens were MTB positive by nested PCR but not by Truenat. Of this group, 3 specimens were CRS− and treatment naive but consequently false positives. On the other hand, 14 of the 30 were MTB positive by Truenat but not nested by PCR. Of this group, all 14 were CRS+ and on antitubercle treatment indicating no false positives

**Table 3:** Comparison of Truenat MTB results with in-house nested PCR results.

	Nested PCR	
Truenat MTB	+	−
+	160	14
−	16	36

Performance estimates of all tests using the CRS as a reference standard are presented in Table 4 As can be seen, the PCR tests have higher sensitivity than smear and culture tests. The IS6110 nested PCR protocol had a PCR inhibition rate of 8.4% (19/226) where the PCR reaction had to be repeated after the DNA was diluted as 1∶1 with sterile water.

**Table 4:** Comparison of all methods against CRS as reference standard.

(N = 226)	CRS vs Others							
	Smear(%)		Culture(%)		Nested(%)		Truenat(%)	
	Pos	Neg	Pos	Neg	Pos	Neg	Pos	Neg
Test Pos	120	71	141	50	173	18	174	17
Test Neg	0	35	0	35	3	32	0	35
Sensitivity	63		74		91		91	
Specificity	100		100		91		100	
PPV	100		100		98		100	
NPV	33		41		64		67	

Liquid culture had an average time to positivity (TTP) of 25 days, in-house nested PCR had a TTP of 7 days (additional 7 days if PCR was inhibited) and the Truenat MTB test had a TTP of approximately 1 hour.

## Discussion

Appliance of molecular methods in routine for diagnosing in developing country like India depends on diverse factors like high cost, rapid and accessibility of skilled personnel to perform the test. Although economical and extensively available, microscopy was nonspecific in our study population and less sensitive than Nucleic acid amplification Test (NAAT) and thus not satisfactory for rapid diagnosis.

**Figure 5:**
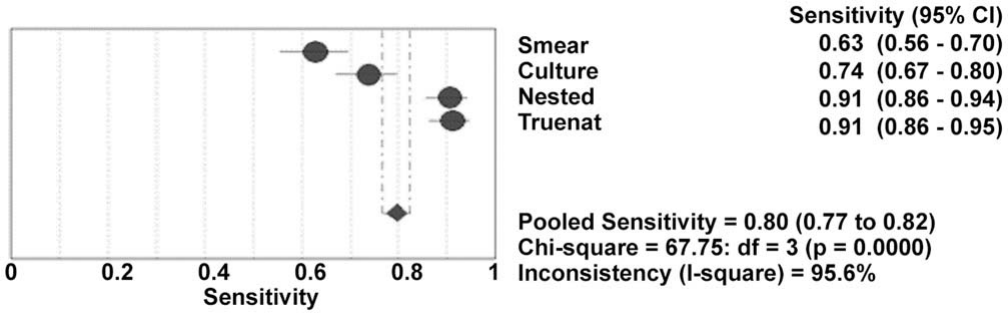
Forest plot for sensitivity values of microbiological and molecular methods. Forest Plot for sensitivity of smear, Culture, Nested and Truenat molecular methods with pooled sensitivity. Performance of molecular methods studies reporting sensitivity. Point estimates of sensitivity estimates from each study are shown as solid circles. Solid lines represent the 95%CI. CI = confidence interval.

**Figure 6:**
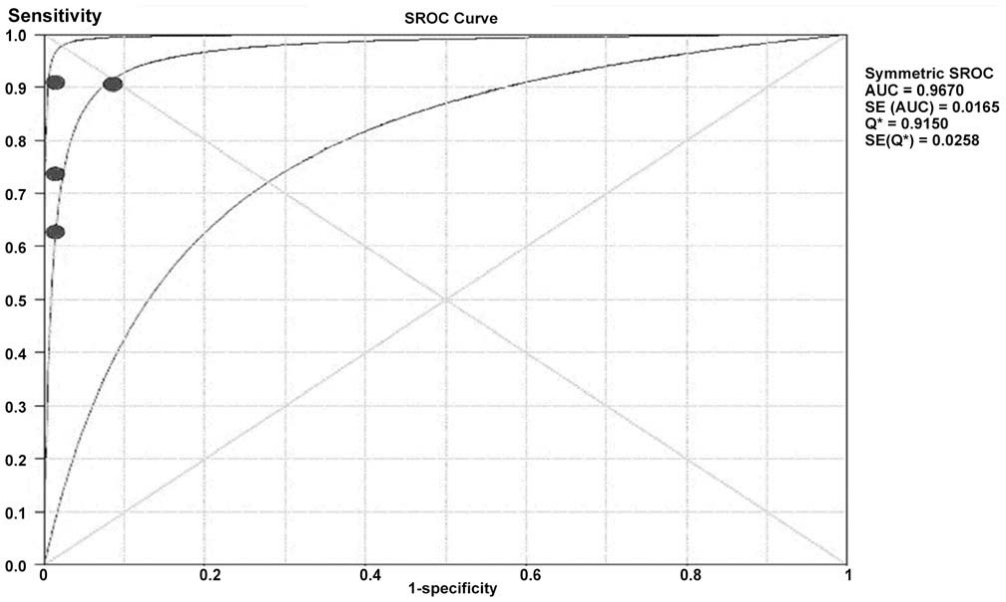
ROC curves for various techniques evaluated in this study. Performance of molecular tests reporting sensitivity and specificity. The curve is the regression line that summarises the overall diagnostic accuracy. Q* is an index defined by the point on the SROC curve where the sensitivity and specificity are equal, which is the point closest to the top-left corner of the ROC space. SROC: summary receiver operating curve; AUC: area under the curve; SE (AUC): standard error of AUC; SE (Q*): standard error of Q* index.

In present study, the Truenat MTB test was found to have sensitivity of 91.1%(CI: 91.1–94.7) and in- house nested PCR have sensitivity of 90.5% (CI: 85.5–94.3). NAAT demonstrated excellent specificities in our study as shown in forest plot and ROC curve (Fig. 5 and Fig. 6) against smear, culture.

### Positivity of culture vs PCR

Pulmonary TB constitutes the major form of TB in clinical practice. In this study, we evaluate the Truenat MTB test for detection of pulmonary TB in near-care settings. As can be seen in Table 3, based on CRS criteria, PCR tests had higher sensitivity compared to smear microscopy and culture. In this study, 50 CRS positive TB cases were culture negative. This could be attributed to unequal distribution of mycobacteria in paucibacillary respiratory specimens. Clumping of micro-organisms is a common problem with mycobacteria, therefore, uniform dispersion in clinical specimens is difficult to obtain [10], [11]. Additionally, during the decontamination procedure, errors such as (i) inappropriate specimen dilution, (ii) accidental aspiration of the pellet when removing supernatant, and (iii) cross-contamination could have resulted in false negative results. Furthermore, the decontamination procedure could have caused a large reduction (80%) of colony forming units (CFU) recovered in cultures [12]. This may have increased the likelihood of culture-negative results among CRS positive specimens.

Many of the culture-negative CRS positive. and on effective treatment cases were accurately identified by the PCR techniques. Discrepancies between conventional mycobacterial culture and PCR-based detection have been previously associated with TB patients who undergo anti-tubercular chemotherapies [13], [14]. PCR can detect DNA from non-viable MTB as a result of anti-tubercular treatment and also viable MTB present in paucibacillary specimens, whereas culture detects viable bacilli.

The load of bacilli require to obtain a positive culture is 100^viable^ bacilli, and lower detection limit of conventional PCR is 10 copies and for real-time PCR it is 6 copies. However as our laboratory is a tertiary care laboratory with a referral bias towards non-responders, most of the patients, 154 out of 226 patients were on anti tubercle treatment Therefore in some instances the bacilli may not be viable. Hence these bacilli may not grow in culture but their DNA could be detected using PCR.

Various nucleic acid amplification tests (NAAT) have been developed for detection of MTB in sputum [15]. In general, different NAAT tests have been found to have positivity in 95–100% of smear and culture positive specimens where as the positivity ranges from 40–60% in smear negative paucibacillary pulmonary disease [15]–[25]. The positivity of the PCR methods evaluated in this study (∼99% positivity in S+C+ specimens and >75% in S−C+ specimens for the in-house nested PCR and Truenat MTB) was in accordance with previously observed values. All of the NAAT studied here have analytical sensitivities for nucleic acid detection quantitatively equivalent to 1–10 mycobacteria [26]–[31].

In terms of sensitivity, assays that detect insertion sequence IS6110 for molecular diagnosis of MTB, such as the one described here, benefit from the fact that IS6110 often presents in high copy numbers.

### Time to positivity (TTP) and ease-of-use

Though culture is inexpensive, the high TTP is an important barrier to rapid detection. In-house PCR protocols such as the IS6110 nested PCR protocol described here, though sensitive; suffer from false positivity due to inherent pitfalls associated with PCR techniques that require post-amplification analysis (carryover contamination between specimens, reagent contamination due to aerosol-based transmission of amplicon). The TTP can be quite long as specimens need to be batched to increase cost-effectiveness. Additionally, PCR inhibition rates can be high (here 8.4%), increasing the TTP by a few days, at the same time increasing the overall cost of PCR-based diagnosis. The Truenat MTB test evaluated in this study had a TTP of approximately one hour, enabling rapid detection of MTB DNA. The optimized sputum processing protocol ensured that PCR inhibitors were removed from the isolated DNA.

Using this test, specimens can be tested without delay as there is no need to wait for additional specimens to be collected and processed. Lyophilized mastermix on chip eliminated the need to wait for reagents to thaw and false positive results due to reagent contamination. The disposable, self-contained chip, designed to be a single-use consumable eliminated the possibility of carryover between specimens. The results are displayed on the screen and can be transmitted via GSM/Wi-Fi/Bluetooth® to a central server or printer. The light weight, portable nature of the devices makes them deployable in peripheral laboratories.

In conclusion, the Truenat MTB test not only has good sensitivity and specificity for the diagnosis of TB but also fits the requirements of the resource-limited health care settings. Large studies are required to obtain better estimates of the Truenat MTB performance.

## References

[1] World Health Organization (2004) World Health Report 2004: Changing history. Geneva: WHO.

[2] World Health Organization (2012) Fact Sheet No. 104: Tuberculosis. Geneva: WHO

[3] Dye C, Watt CJ, Bleed DM, Hosseini SM, Raviglione MC (2005) Evolution of tuberculosis control and prospects for reducing tuberculosis incidence, prevalence, and deaths globally. JAMA 293: 2767–2775. 15941807 10.1001/jama.293.22.2767

[4] Mathema B, Kurepina NE, Bifani PJ, Kreiswirth BN (2006) Molecular Epidemiology of Tuberculosis: Current Insights. Clin Microbiol Rev 19 4: 658–685. 17041139 10.1128/CMR.00061-05 PMC1592690

[5] Kumar KK, Jayaraman R, Narasimha SK, Radhakrishnan RM, Viswanathan S, et al. (2009) PCT Pub. No WO/2009/047805

[6] Kumar KK, Jayaraman R, Narasimha SK, Radhakrishnan RM, Viswanathan S, et al. (2009) Handheld Micro PCR Device. PCT Pub. No.WO/2009/047804

[7] Vadwai V, Boehme C, Nabeta P, Shetty A, Alland D, et al (2011) Xpert MTB/RIF: a new pillar in diagnosis of extrapulmonary tuberculosis? J Clin Microbiol 49 7: 2540–5. 21593262 10.1128/JCM.02319-10 PMC3147857

[8] Deshmukh M, Vadwai V, Nikam C, Ragte T, Shetty A, et al (2012) Is a Composite Reference Standard (CRS) an alternative to culture in assessment and validation of a single tube nested in- house PCR for TB diagnosis. Indian J Tuberc Under publication.

[9] Bossuyt PM, Reitsma JB, Bruns DE, Gatsonis CA, Glasziou PP, et al (2004) Towards complete and accurate reporting of studies of diagnostic accuracy: the STARD initiative. Fam Pract 21: 4–10. 14760036 10.1093/fampra/cmh103

[10] Beige J, Lokies J, Schaberg T, Finckh U, Fischer M, et al (1995) Clinical evaluation of a *Mycobacterium tuberculosis* PCR assay. J Clin Microbiol 33: 90–5. 7535316 10.1128/jcm.33.1.90-95.1995 PMC227886

[11] Clarridge JE III, Shawar RM, Shinnick TM, Plikaytis BB (1993) Large-scale use of polymerase chain reaction for detection of *Mycobacterium tuberculosis* in a routine mycobacteriology laboratory. J Clin Microbiol 31: 2049–56. 8370729 10.1128/jcm.31.8.2049-2056.1993 PMC265694

[12] Yajko DM, Wagner C, Tevere VJ, Kocagöz T, Hadley WK, et al (1995) Quantitative culture of Mycobacterium tuberculosis from clinical sputum specimens and dilution endpoint of its detection by the Amplicor PCR assay. J Clin Microbiol 33: 1944–1947. 7665679 10.1128/jcm.33.7.1944-1947.1995 PMC228308

[13] Bennedsen J, Thomsen VO, Pfyffer GE, Funke G, Feldmann K, et al (1996) Utility of PCR in diagnosing pulmonary tuberculosis. J Clin Microbiol 34: 1407–1411. 8735089 10.1128/jcm.34.6.1407-1411.1996 PMC229033

[14] Traore H, van Deun A, Shamputa IC, Rigouts L, Portaels F (2006) Direct detection of *Mycobacterium tuberculosis* complex DNA and rifampin resistance in clinical specimens from tuberculosis patients by line probe assay. J Clin Microbiol 12: 4384–4388. 10.1128/JCM.01332-06 PMC1698436 17035487

[15] Khandekar P, Amol G, Reddi PP, Banwalikar JN, Shivannavar CT, et al (1994) Evaluation of PCR based test for the detection of *Mycobacterium tuberculosis* in coded sputum specimens. Indian J Med Res 100 167–71. 7851967

[16] Pfyffer GE, Kissling P, John EM, Welsher HM, Salfinger M, et al (1996) Diagnostic performance of amplified *Mycobacterium tuberculosis* direct test with cerebrospinal fluid, other non-respiratory and respiratory specimens. J Clin Microbiol 34: 834–41. 8815093 10.1128/jcm.34.4.834-841.1996 PMC228902

[17] Brisson-Noel A, Gicquel B, Lecossier D, Levy-Frebault V, Nassif X, et al (1989) Rapid diagnosis of tuberculosis by amplification of mycobacterial DNA in clinical specimens. Lancet ii: 1069–71. 10.1016/s0140-6736(89)91082-9 2572798

[18] Hermans PW, Schuitema AR, Van Soolingen D, Verstynen CP, Bik EM, et al (1990) Specific detection of *Mycobacterium tuberculosis* complex strains by polymerase chain reaction. J Clin Microbiol 28: 1204–13. 2116445 10.1128/jcm.28.6.1204-1213.1990 PMC267906

[19] Sjobring U, Mockkenberg M, Andrensen AB, Miomer H (1990) Polymerase chain reaction for the diagnosis of *Mycobacterium tuberculosis* . J Clin Microbiol 28: 2200–4. 2121782 10.1128/jcm.28.10.2200-2204.1990 PMC268147

[20] Eisenach KD, Gifford MD, Bates JH, Crawford JT (1991) Detection of *Mycobacterium tuberuculosis* in sputum specimens using a polymerase chain reaction. Am Rev Respir Dis 144 1160–3. 1952448 10.1164/ajrccm/144.5.1160

[21] Reddi PP, Talwar GP, Khandekar PS (1993) Molecular cloning and characterization of contiguoly located repetitive and single copy sequences of *Mycobacterium tuberculosis*: development of PCR based diagnosis assay. Int J Lepr 61: 227–35. 8371032

[22] Baevis KG, Litchy MB, Jungkind DL, Giger O (1995) Evaluation of amplicor PCR direct detection of *Mycobacterium tuberculosis* from sputum specimens. J Clin Microbiol 33: 2582–6. 8567886 10.1128/jcm.33.10.2582-2586.1995 PMC228532

[23] Jonas V, Alden MJ, Curry JI, Kamisango K, Knott CA, et al (1993) Detection and identification of *Mycobacterium tuberculosis* directly from sputum sediments by amplification of rRNA. J Clin Microbiol 33: 2410–6. 10.1128/jcm.31.9.2410-2416.1993 PMC265770 8408564

[24] Miller N, Hernandez SG, Cleary TJ (1994) Evaluation of gene probe amplified *Mycobacterium tuberculosis* direct test and PCR for direct detection of *Mycobacterium tuberculosis* in clinical specimens. J Clin Microbiol 32: 393–7. 8150948 10.1128/jcm.32.2.393-397.1994 PMC263042

[25] Shah JS, Liu J, Buxton D, Hendricks A, Robinson L, et al (1995) Q-beta replicase amplified assay for detection of *Mycobacterium tuberculosis* directly from clinical specimens. J Clin Microbiol 33: 1435–41. 7650163 10.1128/jcm.33.6.1435-1441.1995 PMC228191

[26] Eisenach KD, Sifford MD, Bates JH, Cave MD, Crawford JJ (1991) Detection of *Mycobacterium tuberculosis*. in sputum samples using a polymerase chain reaction. Am Rev Respir Dis 144: 1160–3. 1952448 10.1164/ajrccm/144.5.1160

[27] Kent PT, Kubica GP (1985) Public health mycobacteriology: a guide for the level three laboratory. Atlanta: Centers for Disease Control:36.

[28] Williams DL, Gilles TP, Dupree WG (1995) Ethanol fixation of sputum sediments for DNA-based detection of *Mycobacterium tuberculosis* . J Clin Microbiol 33: 1558–61. 7650186 10.1128/jcm.33.6.1558-1561.1995 PMC228215

[29] Longo MC, Berninger MS, Hartley JL (1990) Use of uracil DNA glycosylase to control carry-over contamination in polymerase chain reactions. Gene 93: 125–8. 2227421 10.1016/0378-1119(90)90145-h

[30] Beige J, Lokies J, Schaberg T, Finckh U, Fischer M, et al (1995) Clinical evaluation of a *Mycobacterium tuberculosis* PCR assay. J Clin Microbiol 33: 90–5. 7535316 10.1128/jcm.33.1.90-95.1995 PMC227886

[31] Kellogg DE, Rybalkin I, Chen S, Mukhamedova N, Vlasik T, et al (1994) TaqStart Antibody: “hot start” PCR facilitated by a neutralizing monoclonal antibody directed against Taq DNA polymerase. Biotechniques 16: 1134–7. 8074881

